# Embryo transfer following IVF alters susceptibility to metabolic phenotypes in male mouse offspring compared to naturally conceived offspring

**DOI:** 10.1530/RAF-26-0031

**Published:** 2026-05-28

**Authors:** Katharina Laurent, Sandra Hoffmann, Susan Marschall, Kerstin B Richter, Raffaele Teperino, Martin Hrabě de Angelis, David A Skerrett-Byrne, Johannes Beckers

**Affiliations:** ^1^ Institute of Experimental Genetics, Helmholtz Zentrum München, German Research Center for Environmental Health Neuherberg, Neuherberg, Germany; ^2^ German Center for Diabetes Research (DZD) Neuherberg, Neuherberg, Germany; ^3^ German Mouse Clinic, Helmholtz Zentrum München, German Research Center for Environmental Health (GmbH), Neuherberg, Germany; ^4^ Chair of Experimental Genetics, TUM School of Life Sciences, Technische Universität München, Freising, Germany; ^5^ Infertility and Reproduction Research Program, Hunter Medical Research Institute, New Lambton Heights, New South Wales, Australia; ^6^ School of Biomedical Sciences and Pharmacy, College of Health, Medicine and Wellbeing, The University of Newcastle, Callaghan, New South Wales, Australia; ^7^ INFRAFRONTIER ERIC, Neuherberg, Germany

**Keywords:** IVF, metabolism, offspring

## Abstract

The aim of this study is to highlight potential differences between embryos conceived naturally and those conceived via *in vitro* fertilisation (IVF). While the current scientific literature generally assumes that differences between these two modes of conception exist, many studies do not provide detailed phenotypic data, e.g. bodyweight, blood glucose and other observational metabolic health parameters. In addition, comparisons are often complicated by variations in choosing different strains of foster mothers to carry embryos. By addressing these limitations, our short summary and overview provide a well-defined comparison within a controlled experimental setup including a high-fat diet feeding period as metabolic challenge. We make the resulting data publicly available to offer a reference point for researchers using a specific mouse strain as fosters and to improve transparency regarding the specific differences in metabolic health observed between natural conception and IVF in this context.

## Main text

One in six European couples experience infertility, and global infertility rates continue to rise (Skerrett-Byrne *et al.* 2025). Consequently, *in vitro* fertilisation (IVF) is increasingly employed as an assisted reproductive method to circumvent these growing infertility issues. It is well established that IVF-derived offspring in both humans and mice exhibit differences compared to naturally conceived individuals (Guo *et al.* 2017, Rhon-Calderon *et al.* 2025). However, outcomes reported in animal studies are highly dependent on strain and experimental setup, highlighting substantial unresolved uncertainty in the literature. Although many experimental designs use IVF to control for *in utero* effects and other confounders, the procedure itself introduces biological perturbations whose associated comorbidities must be carefully considered.

In this study, we comprehensively examined metabolic phenotypes in C57BL/6N mice offspring derived from natural conception (NC) and embryo transfer following IVF (embryo transfer; ET). Detailed methods are attached in the supplementary material (S1 (see section on Supplementary materials given at the end of the article)). CD1 foster mothers, commonly used due to their robust maternal behaviour, served as recipients for IVF-derived embryos. We used protocols of the European Mouse Mutant Archive (EMMA) for sperm collection and analysis, IVF and embryo transfer, publicly available at https://www.infrafrontier.eu/emma/cryopreservation-protocols/. Phenotypic differences between NC- and ET-derived mice may originate from the IVF procedure, the embryo transfer or embryo culture conditions and *in utero* development in foster mothers as well as from differences in milk availability and differences in rearing behaviour.

**Figure 1:**
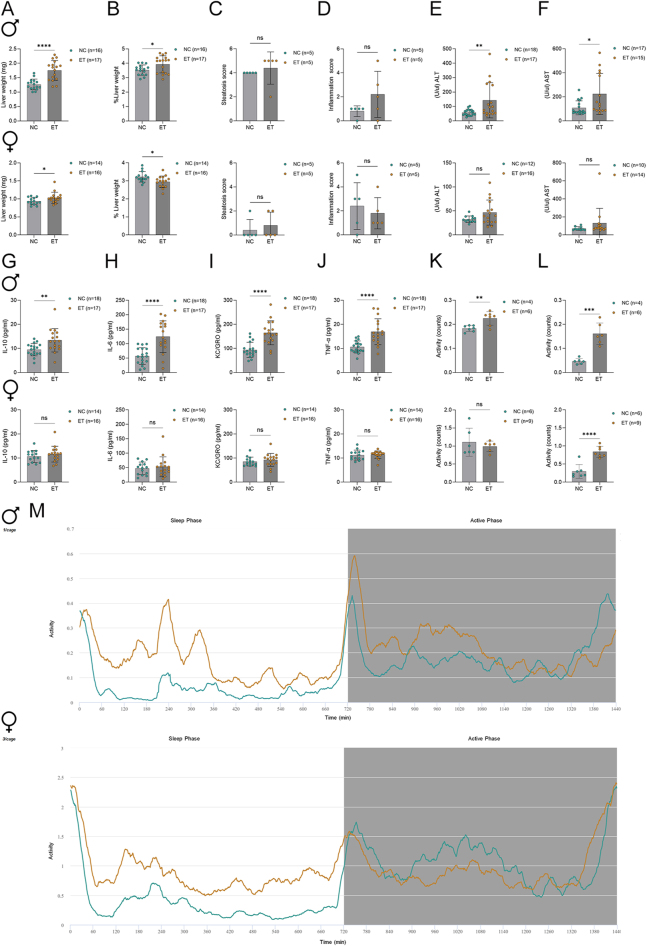
Liver, inflammation and activity assessment of offspring derived from NC and embryo transfer (ET). All tests were performed after organ withdrawal at 16 weeks of age, and exceptional activity measurement was performed during the 5th week of the HFD challenge. (A) Liver weight of female and male NC and ET offspring and (B) liver weight expressed as a percentage of bodyweight. (C) Histological steatosis scores and (D) histological inflammation scores in livers from of NC and ET offspring. (E) Plasma levels of alanine aminotransferase (ALT) and (F) aspartate aminotransferase (AST) in NC and ET offspring. Circulating levels of cytokines (G) IL-10 and (H) IL-6, (I) keratinocyte chemoattractant (KC)/human growth-regulated oncogene (GRO) and (J) TNF-alpha in NC and ET offspring. (K) Activity counts during the sleep and (L) active phases of female and male NC and ET offspring. (M) Visualisation of the average locomotor activity observed during the course of a HFD challenge. Data are presented as mean with SD, and statistical significance is indicated as *P*-values: *<0.05, **<0.01, ***<0.001, ****<0.0001.

At 9 weeks of age, the offspring was challenged with high-fat diet (HFD) until organ withdrawal at 16 weeks of age, to enhance the development of potential metabolic phenotypes. All tests were performed after the HFD challenge at 15 weeks of age, exceptional activity measurement was performed during the 5th week of the HFD challenge, and body weight was measured weekly. Additionally, blood glucose was measured at 9, 12, 15 and 16 weeks of age. Beyond the known increases in body weight and glucose metabolism in IVF-conceived offspring (Fig. S2 A, B, C, D, E, F) (Rhon-Calderon *et al.* 2025), we identified marked alterations in liver parameters (Fig. 1A, B, C), activity patterns (Fig. 1K, L, M) and inflammation markers (Fig. 1D, E, F, G, H, I, J). The litter size was just slightly increased in NC-derived mice (Fig. S2G), and no differences in sex distribution was observed (Fig. S2H). Strikingly, there appears to be a sex-specific metabolic change, with male IVF offspring more severely affected compared to their NC male counterparts. However, female IVF offspring exhibited a significantly increased body composition (% of fat mass) after the HFD challenge (Fig. S2D). This increased body fat could point towards a higher susceptibility to develop obesity. The observed elevation in alanine aminotransferase (ALT), aspartate aminotransferase (AST) and liver weight, together with increased pro-inflammatory cytokines (TNF-α, IL-6, keratinocyte chemoattractant (KC)/human growth-regulated oncogene (GRO)) and the compensatory anti-inflammatory IL-10 (Fig. 1A, B, C, D, E, F, G, H, I, J), suggests that male IVF offspring experience hepatic and systemic inflammatory stress (Al-Qahtani *et al.* 2024). The absence of these changes in female offspring points to possible sex-dependent mechanisms, potentially mediated by hormonal or epigenetic factors (Aljabali *et al.* 2024). Hyperactivity was observed in both sexes of the IVF offspring during their habitual sleep phase (Fig. 1L), which points towards a potential disruption of circadian regulatory mechanisms. However, this could also have other causes, e.g. stress response or metabolic changes.

In conclusion, these findings underscore the potential impact of IVF and related confounding factors on offspring’s metabolic health and emphasise the importance of carefully evaluating reproductive methods and their potential long-term consequences in experimental animal models.

## Supplementary materials



## Declaration of interest

The authors declare no conflicts of interest. DA Skerrett-Byrne is an Associate Editor of *Reproduction & Fertility* and was not involved in the peer review or editorial process for this paper, on which he is listed as an author.

## Funding

This work was funded by National Health and Medical Research Council (NHMRC) Emerging Leadership Fellowship (APP2034392) awarded to DASB. Part of this work was funded by grants from DZD e.V. to JB and MHdA.

## Author contribution statement

KL, DASB and JB conceived the study; KL, SM, SH and KR performed investigation; KL, DASB and JB performed formal analysis; MHA, RT and JB provided resources; KL, DASB and JB wrote the original manuscript; KL, DASB, SM, SH, KR, RT, MHA and JB reviewed and edited the manuscript; KL, DASB and JB performed visualisation; DASB and JB acquired funding; DASB, MHA and JB supervised the study.
