# Supplementary material for: Embryo transfer following IVF alters susceptibility to metabolic phenotypes in male mouse offspring compared to naturally conceived offspring
Source: Reprod Fertil. 2026 May 28;7(2):RAF260031. doi: 10.1530/RAF-26-0031 (PMC13232599; doi:10.1530/RAF-26-0031)

Methods S1. Detailed information about phenotypic procedures and statistics.

METHODS

All mice were housed in digital ventilated cages on a 12-h light/12-h dark cycle at constant temperature (22 ± 1 °C) and under controlled humidity. Mice had ad libitum access to water and food. Mouse experiments were conducted in compliance with the Federation of European Laboratory Animal Science Association (FELASA) protocols. All mouse experiments were performed according to ROB-55.2-2532.Vet_02-23-59 approved by the Regierung von Oberbayern.

In vitro fertilization and embryo transfer

In vitro fertilization (IVF) was conducted with gametes of C57BL/6N mice at the age of 17-18 weeks. In parallel animals were mated at the same age. Female oocyte donors were stimulated with 7.5 U of pregnant mare serum gonadotropin (PMSG) and 7.5 U of human chorionic gonadotropin (hCG) 64 and 14 hours prior to oocyte collection, respectively. Fresh sperm was obtained from the cauda epididymis and cultured in preincubation medium for 1 hour to allow capacitation. Oocytes were collected from the ampulla and co-cultured with 5 µL of capacitated sperm in human tubal fluid (HTF) medium at 37 °C and 5% CO2. After incubation for 4-6 hours, fertilized oocytes were washed in HTF to remove sperm and cumulus cells, transferred into potassium simplex optimization medium (KSOM) medium and incubated overnight at 37 °C and 5% CO2. In order to obtain foetuses and living offspring, 2-cell embryos were subjected to embryo transfer. Therefore, 2-cell embryos were transferred into pseudo-pregnant foster mothers of the CD-1 strain that have been mated with vasectomized males on the previous day and displayed a vaginal plug. CD-1 females were anaesthetized with a mixture of ketamin (100 mg/kg) and xylazin (16 mg/kg) before surgery and embryos were transferred bilaterally into the oviducts through two minor incisions. Postsurgical pain management was ensured by oral application of Novalgin (200 mg/kg). We used protocols of the European Mouse Mutant Archive (EMMA) for sperm collection and analysis, IVF and embryo transfer, publicly available under: https://www.infrafrontier.eu/emma/cryopreservation-protocols/.

Offspring were maintained on an identical dietary regimen. Standard chow was fed until 9 weeks of age. At 9 weeks of age, they were switched to a high-fat diet (HFD) for 6 weeks as a metabolic challenge.

DVC

Spontaneous locomotor activity and behaviour were monitored using the DVC™ (Digital Ventilated Cage) home cage monitoring system (Tecniplast, Buguggiate, Italy). Mice were housed in standard individually ventilated cages equipped with the DVC™ sensor system under controlled temperature and humidity conditions with a 12 h light/dark cycle. Food and water were available ad libitum.

The DVC™ system continuously recorded animal activity through capacitance-based sensing of movement within the cage floor plate. Activity data were collected and processed using the manufacturer's software. Parameters including locomotor activity patterns and circadian rhythmicity were extracted.

Litter Size

From animals derived with natural conception there were in total 5 litters to analyse, for embryo transfer followed by IVF derived offspring 7 litters. The litter size of NC derived offspring was slightly increased. The sex distribution was evenly between both groups.

Analysis

For NC offspring 14 females and 18 males were analysed, depending on the test the n-numbers could vary, indicated in the figure panels. The tests were performed per animal. For the activity analysis we monitored 4 male animals of NC and 6 male animals of ET analysed over 7 following days during the 5th week of the HFD challenge. For the females the animals were group caged in groups of 3, therefore 6 animals of NC and 9 animals of ET were analysed. All tests were performed after the HFD challenge at 15 weeks of age, exceptionally activity measurement was performed during the 5th week of the HFD challenge, as well as body weight was measured weekly.

Glucose tolerance test. Mice were fasted for 4 h during light phase before administration of 2 g of glucose per kg body weight by intraperitoneal injection. Blood was collected from the tail vein at 0, 15, 30, 60 and 120 min and blood glucose concentrations were measured using a Contour Bayer glucometer. The test was performed at 15 weeks of age.

Fasting blood glucose. Mice were fasted for 4h during light phase. Blood was collected from the teil vein and blood concentrations were measured using a Contour Bayer glucometer. The test was performed at 9, 12, 15 and 16 weeks of age.

Statistical analysis of physiological parameters from mice GraphPad Prism v.10.4.1 was used to analyse physiological data from mice. Graphs are shown with SD. Unpaired t-test for all bar graphs. Mixed models with multiple testing correction for bodyweight, glucose tolerance test and blood glucose measurement. *$p \leq 0.05$, **$p \leq 0.01$, *** $p \leq 0.001$.

Figure S2. Phenotypic characterisation of offspring derived from natural conception (NC) and embryo transfer (ET). All the tests were performed after the HFD challenge at 15 weeks of age, exceptionally body weight was measured weekly. (A) Body weight of female and male NC and ET offspring at weaning and (B) during the course of a high-fat diet (HFD) challenge initiated at 9 weeks of age. (C) Body composition of NC and ET offspring prior to and (D) post HFD exposure. (E) Glucose tolerance test was performed at 15 weeks of age, whilst (F) blood glucose measurement was taken at 9, 12, 15 and 16 weeks in NC and ET offspring. (G) Litter size from NC and ET pregnancies and (H) sex distribution of offspring across groups. Data are presented as mean with SD and statistical significance is indicated as *p*-values: *<0.05, ****<0.0001

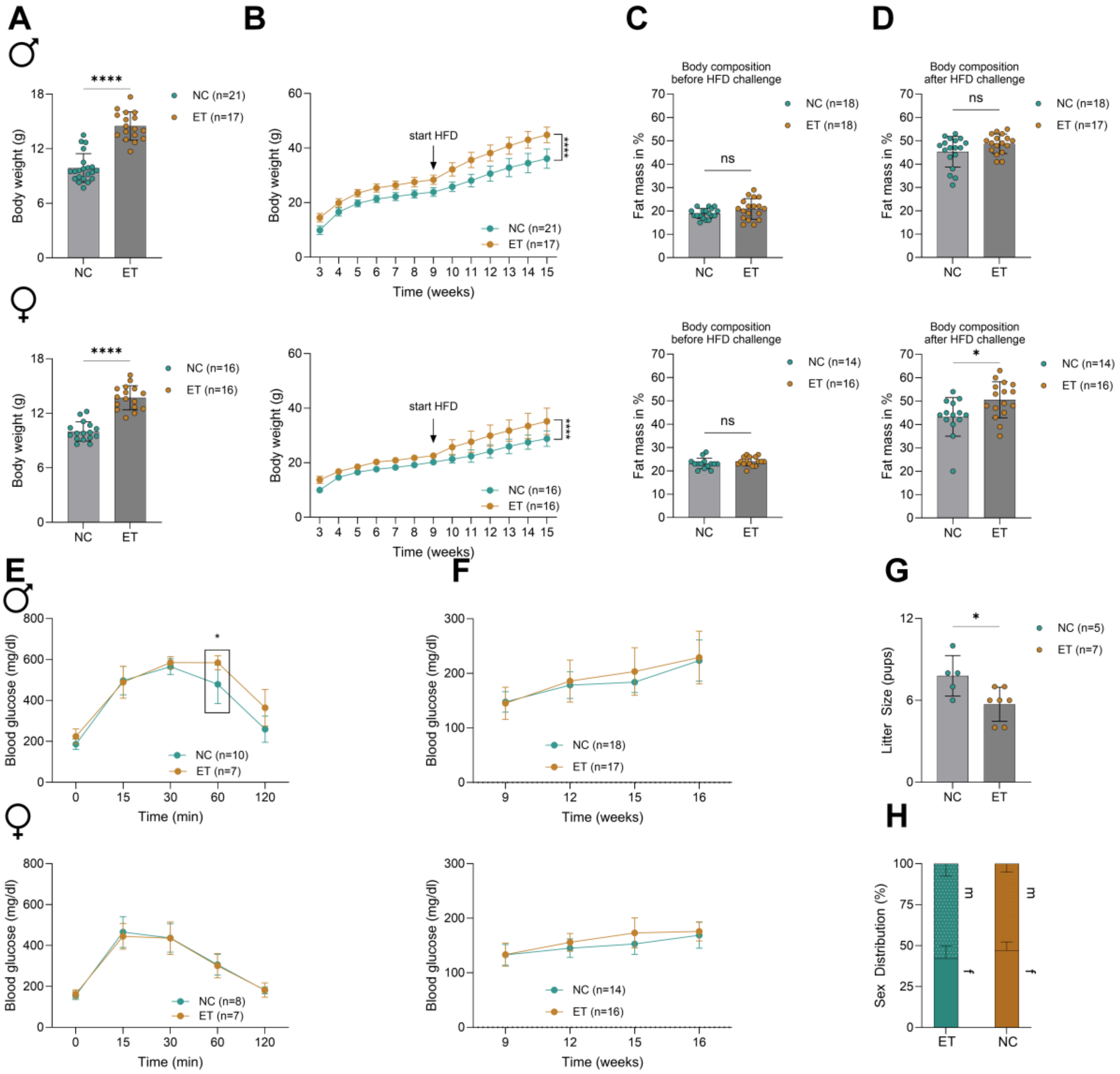

Supplement: Supplementary file 1 [file supplementary_materials.pdf]
